# *FAAH* rs324420 Polymorphism in People With Opioid and Methamphetamine Use Disorders: Psychological Associations

**DOI:** 10.1002/brb3.71477

**Published:** 2026-05-14

**Authors:** Özlem Erekli‐Arat, Meltem Tepe, Selin Özkan‐Kotiloğlu, Mustafa Danışman, Dilek Kaya‐Akyüzlü

**Affiliations:** ^1^ Institute of Forensic Sciences Ankara University Ankara Türkiye; ^2^ Faculty of Science and Art, Department of Molecular Biology and Genetics Kırşehir Ahi Evran University Kırşehir Türkiye; ^3^ Ankara Training and Research Hospital AMATEM Clinic Ankara Türkiye

**Keywords:** fatty acid amide hydrolase (FAAH), methamphetamine, opioids, PCR‐RFLP, rs324420 polymorphism

## Abstract

**Purpose:**

The global increase in the prevalence of substance use disorders (SUD) has resulted in higher mortality rates. While heroin use has decreased or stabilized, methamphetamine use is increasing, and the combined use of opioids and methamphetamine is becoming more common. FAAH is an enzyme that hydrolyzes endocannabinoid substances and plays a role in neurobehavioral processes. The aim of this study was to investigate the effect of the *FAAH* rs324420 polymorphism on SUD.

**Method:**

The study included 562 individuals who used methamphetamine (MUD, *n* = 148), opioids (OUD, *n* = 114), or both methamphetamine and opioids (OMCU, *n* = 150), as well as 150 healthy volunteers. The *FAAH* rs324420 polymorphism was analyzed by PCR‐RFLP. All participants completed the Barratt Impulsiveness Scale‐11 (BIS‐11), Clinical Opiate Withdrawal Scale (COWS), Substance Craving Scale (SCS), Beck Anxiety Inventory (BAI), and Beck Depression Inventory‐II (BDI‐II).

**Finding:**

The frequencies of the variant allele (A) were 21%, 13%, 16%, and 19% in OUD, MUD, OMCU, and controls, respectively. Genotype frequencies in all four groups were in Hardy–Weinberg equilibrium (*p* > 0.05). The CA genotype significantly reduced the risk of the disease compared to the reference genotype (OR = 0.578, 95% CI:0.343–0.973, adjusted *p* = 0.039). Craving scale score was higher in individuals who concurrently used opioids and methamphetamine (20.0; 13.0–28.0) than in those who only used opioids (18.0; 14.0–28.0) (*p* = 0.009).

**Conclusion:**

This study showed that the rs324420 CA genotype of the *FAAH* gene may confer a protective effect against methamphetamine use disorder. Future studies with larger sample sizes and additional phenotypic traits are recommended to further explore these findings in other populations.

## Graphical abstract

This study explored the association between the *FAAH* rs324420 polymorphism and substance use disorders in individuals with methamphetamine, opioid, and combined use. The rs324420 CA genotype was linked to a reduced risk of substance use disorder, while higher craving scores were observed in individuals with combined opioid–methamphetamine use.

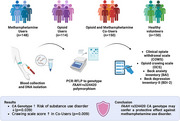

## 1 Introduction

According to the UNODC (2023), the number of people using psychoactive substances worldwide has reached 296 million and is growing rapidly. It is estimated that 1 in 13 individuals aged 15–64 use substances, and more than 1% have a substance use disorder (SUD) (UNODC 2023). As is the case around the world, substance use among young people in Turkey is increasing rapidly. Recently, methamphetamine use and related deaths have increased. According to the TUBIM 2023, of 246 SUD‐related deaths, 56.9% (*n* = 140) were attributed to methamphetamine, 27.2% (*n* = 67) to opioids, and 18.7% (*n* = 46) to ecstasy. Additionally, methamphetamine was detected in 52.3% of 153 deaths involving polysubstance use, and it was the cause of 64.5% of 93 single‐drug deaths. Furthermore, 37.4% of individuals receiving treatment for SUD used heroin (compared to 43% in 2021), while 37.8% used methamphetamine (up from 25.6% in 2021) (TUBIM 2023). Studies show that heroin use is decreasing or stable; however, methamphetamine use is increasing. Recently, the combined use of heroin and methamphetamine has become more common in polysubstance use (Cicero et al. 2020; Jones et al. 2020).

The co‐use of opioids and methamphetamine, often referred to as the “fourth wave of the opioid crisis,” characterized by polysubstance use, is an emerging trend (Ware et al. 2021). The term “speedball” is widely used among users to describe a mixture of heroin and methamphetamine. But why do people use heroin and methamphetamine simultaneously? The proportions of heroin and methamphetamine in speedball vary depending on individuals' opioid tolerance, social status, and personal preferences (Ondocsin et al. 2023). When opioid receptors are maximally occupied, the use of additional opioids has little effect. However, the combined use of stimulants and opioids increases dopamine release, producing a greater synergistic effect than either substance alone (Pattison et al. 2012). Generally, reasons for co‐using methamphetamine and heroin include the desire to relieve opioid withdrawal symptoms, the perception of methamphetamine as a cheaper alternative to heroin, the intention to avoid or protect against overdose, the pursuit of synergistic highs, and the perception of methamphetamine as a safer option to achieve sudden highs (Lopez et al. 2021). In one study, three reasons were identified for combining heroin with methamphetamine: the pleasure derived from substance use for relaxation and “internal use,” “opioid substitution” to limit and manage heroin use, and “unintentional/ordinary/occasional” methamphetamine use. Heroin was the primary substance prioritized by most participants. Some reported mixing heroin and methamphetamine to enhance the synergistic effect, while others noted that one substance's effect was diminished by the other (Ondocsin et al. 2023).

The endocannabinoid system consists of two cannabinoid receptors (CB1 and CB2), the endogenous ligands arachidonoylethanolamine/anandamide (AEA) and 2‐arachidonylglycerol (2‐AG), and the endocannabinoid‐hydrolyzing enzymes FAAH and MAGL (monoacylglycerol lipase) (Bifulco 2009; di Marzo et al. 2004). This system affects several bodily systems, including the central nervous, cardiovascular, gastrointestinal, reproductive, and immune systems (Gündüz 2018). Blocking the endocannabinoid system has been shown to cause depressive behavior in animals (Hill and Gorzalka 2005; Martin et al. 2002), and can also lead to stress and anxiety, thereby increasing the likelihood of substance use disorders (Hill et al. 2008; Grant et al. 2012). FAAH, a key regulator of endocannabinoid signaling, is an enzyme that hydrolyzes anandamide into fatty acid and ethanolamine (Chiang et al. 2004; Deutsch and Chin 1993; Deutsch et al. 2002). Anandamide may act as a feedback mechanism to regulate dopamine release and the effects of receptor activation (Vinklerová et al. 2002). FAAH is considered a potential therapeutic target for several disorders (Santoso and de Ridder 2023). Animal studies suggest that FAAH enzyme inhibitors have antidepressant effects, and that anandamide regulates mood, making FAAH a promising target for antidepressant drugs (Gobbi et al. 2005). The role of FAAH in neurobehavioral processes and withdrawal symptoms has been highlighted in several studies (Zhang et al. 2020). FAAH, a key regulator of endocannabinoid signaling, is also involved in pain perception, energy metabolism, appetite regulation, and inflammation, and has been implicated in substance use disorders and the reward system (Santoso and de Ridder 2023).

The *FAAH* gene is located on chromosome 1p33. It encodes a protein consisting of 579 amino acids and contains 15 exons and 14 introns. Various polymorphisms found in this gene lead to altered mRNA stability, changes in transcription rates, or reduced activity of the encoded protein (López‐Moreno et al. 2012). The rs324420 polymorphism (Pro129Thr or C385A) is a variant located in exon 3 of the *FAAH* gene (Deutsch and Chin 1993). This polymorphism causes functional abnormalities in the endogenous cannabinoid system, leading to physical dependence and alterations in the brain's reward system by altering central nervous system signaling lipids (Sipe et al. 2002; Zhang et al. 2020). Several studies have reported that the rs324420 polymorphism in the *FAAH* gene is associated with addiction to cocaine, alcohol, marijuana, heroin, nicotine, and other substances (Chiang et al. 2004; López‐Moreno et al. 2012; Sipe et al. 2002). Another study found an association between the *FAAH* rs324420 polymorphism and methamphetamine use disorder (Zhang et al. 2020). In addition, recent studies and meta‐analyses have emphasized the potential role of FAAH in the neurobiology of methamphetamine use disorder (Guerin et al. 2021; Guerin et al. 2024; Khan et al. 2025). However, to our knowledge, the effect of the *FAAH* gene polymorphisms has not yet been studied in individuals who co‐use heroin and methamphetamine. In the present study, the effect of the *FAAH* rs324420 polymorphism on opioid‐methamphetamine co‐use, as well as its impact on impulsivity, depressive symptoms, and anxiety symptoms in individuals was assessed.

## 2 Materials and Method

In this study, 114 individuals diagnosed with opioid use disorder (OUD), 148 individuals diagnosed with methamphetamine use disorder (MUD), 150 individuals diagnosed with both opioid and methamphetamine use disorders (OMCU), and 150 healthy volunteers with no history of substance use were included for comparison. Participants with a substance use disorder (SUD) were evaluated and diagnosed by experienced psychiatrists at the Alcohol and Substance Addiction Treatment and Training Centre (AMATEM) at Ankara Training and Research Hospital, according to DSM‐5 criteria. Inclusion criteria for the study were: (1) being between 18 and 65 years of age, (2) being diagnosed with a substance use disorder (opioid and/or methamphetamine) according to DSM‐5 criteria, and (3) choosing to receive inpatient or outpatient treatment. Exclusion criteria included: (1) use of substances other than methamphetamine or heroin (excluding cigarettes), (2) presence of major cognitive or psychotic disorders, assessed via clinical interviews based on DSM‐5 criteria, and (3) neurological diseases affecting the central nervous system.

The study was approved by the local ethics committee (approval number: I03‐109‐22) on March 10, 2022. Each participant who met the inclusion and exclusion criteria completed a demographic information form, which included details such as sex, height and weight, age, marital status, education, housing status, occupation, military service status, school disciplinary history, military disciplinary history, prison history, probation history, family history of substance use, age of onset of first substance use, methods of substance use, and specific methods and frequency of combined heroin and methamphetamine use, daily cigarette consumption, treatment type (inpatient or outpatient), and history of unsuccessful buprenorphine (BUP) treatment. Height and weight were recorded as part of the socio‐demographic and physical characteristics of the participants to provide a comprehensive description of the study population and to examine potential differences that could confound the analyses. In addition, individuals with SUD completed the Barratt Impulsivity Scale‐11 (BIS‐11), the Clinical Opiate Withdrawal Scale (COWS), the Opioid Craving Scale (OCS), the Beck Anxiety Inventory (BAI), and the Beck Depression Inventory‐II (BDI‐II). Previous studies have demonstrated the validity and reliability of the Turkish versions of these scales (Canan et al. 2015; Evren et al. 2014; Güleç et al. 2008; Hisli 1989; Ulusoy et al. 1998). Craving and withdrawal symptoms were evaluated only for opioids, as validated and widely used scales are available for opioid withdrawal and craving, while no standardized or validated scales exist for methamphetamine withdrawal or craving in Turkish clinical practice.

### 2.1 Collection of Blood Samples and DNA Isolation

The principles of the Declaration of Helsinki were adhered to during blood sample collection. A sample of venous blood was taken from each participant and placed into an EDTA tube. Pure genomic DNA was isolated from 200 µL of blood samples in the EDTA tubes using the Thermo Scientific GeneJET Whole Blood Genomic DNA Purification Mini Kit, following the manufacturer's instructions. The yield and quality of the purified DNA were assessed using a NanoDrop spectrophotometer (Thermo Fisher Scientific, USA). DNA concentration was determined by measuring absorbance at 260 nm, and purity was evaluated using A260/A280 ratios.

### 2.2 Use of PCR and RFLP Techniques

To identify the *FAAH* rs324420 polymorphism, forward (F: 5‐′TGTTGCTGGTTACCCCTCTC‐3′) and reverse (R: 5′‐CCCAAAATGACCCAAGATGC‐3’) primers (Sentebiolab) were used, and the region containing the relevant polymorphism was amplified using the polymerase chain reaction (PCR) method.

**FIGURE 1:**
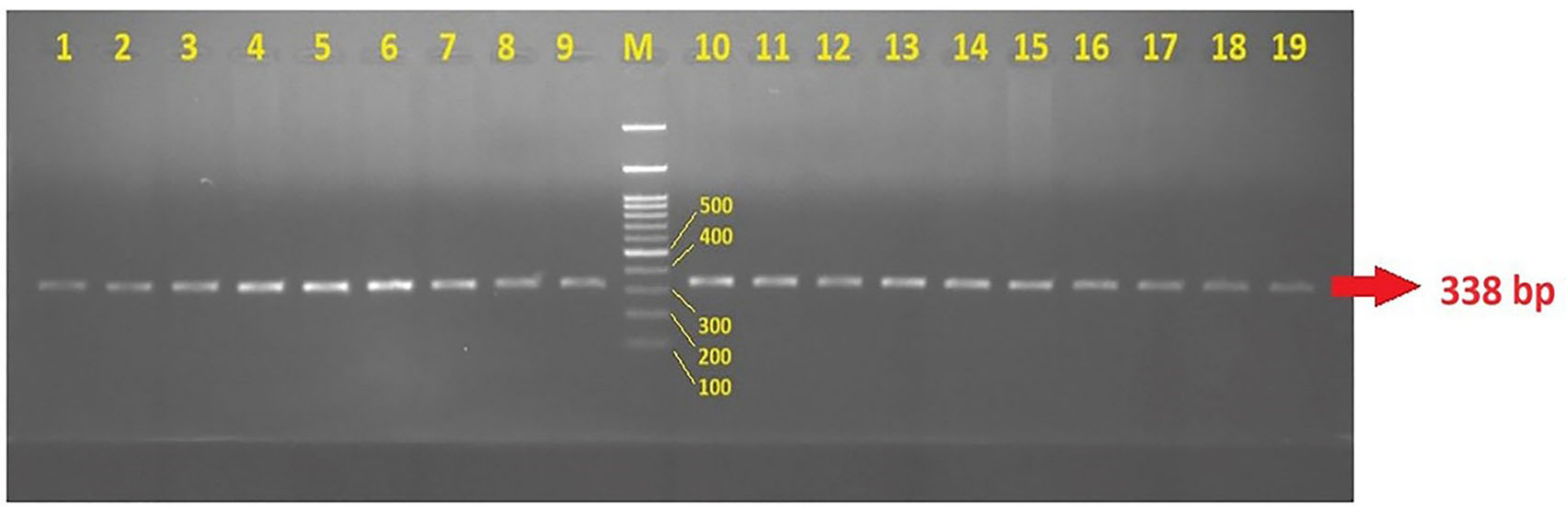
Agarose gel electrophoresis image of the 338 bp PCR product of the *FAAH* rs324420 gene polymorphism (M: DNA ladder; 1–19: 338 bp PCR product).

**FIGURE 2:**
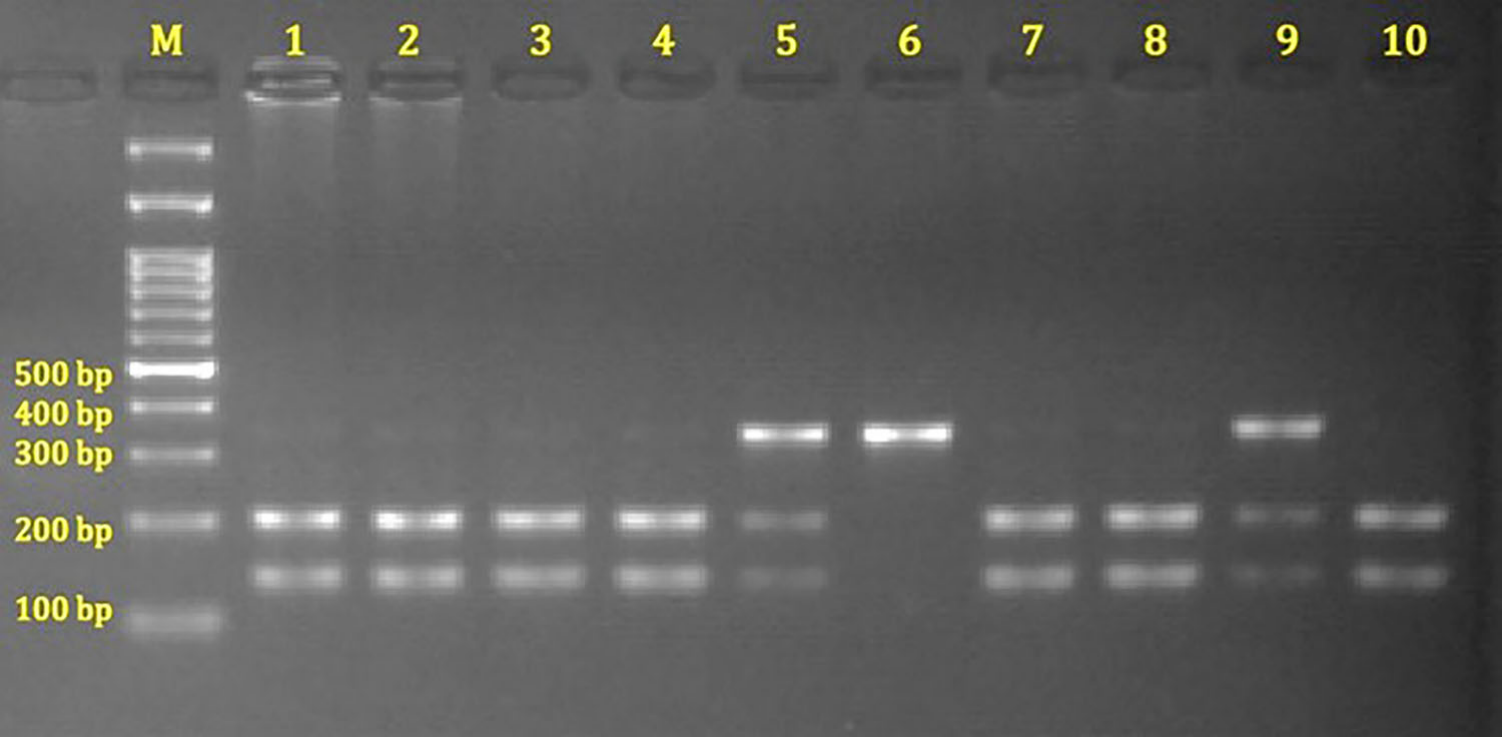
Agarose gel electrophoresis image of PCR products of the rs324420 polymorphism in the *FAAH* gene. M: DNA ladder; lines 1–4, 7, 8, 10: CC (200 bp and 138 bp); lines 5, 9: CA (338 bp, 200 bp, and 138 bp); line 6: AA (338 bp).

PCR amplification for the *FAAH* rs324420 polymorphism was performed with a final volume of 25 µL, containing 2 µL of 200 ng genomic DNA, 2.5 µL of 10X Tango Buffer, 0.4 µL of 10 mM dNTP, 1.25 µL of 25 mM MgCl_2_, 1 µL of 10 pmol F and R primers, and 0.125 µL of 5U Taq polymerase. The mixture was amplified using a thermal cycler (Techne Tc512). The PCR program included an initial denaturation step at 95°C for 5 min (1 cycle), followed by 35 cycles of denaturation at 95°C for 30 s, annealing at 66°C for 30 s, and extension at 72°C for 30 s, with a final extension step at 72°C for 5 min (1 cycle). The resulting 338 bp PCR products (Figure 1) were digested with the *Sty1* restriction enzyme (Thermo) using the restriction fragment length polymorphism method and incubated overnight at 37.0°C. The digested oligonucleotides were separated by 2% agarose gel electrophoresis and analyzed using a gel imaging system. The presence of 200 and 138 bp bands indicated the CC genotype; bands at 338, 200, and 138 bp indicated the CA genotype; and a single 338 bp band indicated the AA genotype (Figure 2). To confirm the accuracy of the RFLP results, 10 samples from each genotype were randomly selected and validated by DNA sequencing.

### 2.3 Statistical Analyses

The results of the study were statistically analyzed comparatively. Statistical analyses were performed using the Statistical Package for the Social Sciences (SPSS, V.25, Chicago, USA). The Hardy–Weinberg equilibrium (p^2^+2pq+q^2^ = 1) was assessed based on the genotype and allele frequencies of the *FAAH* rs324420. When analyzing in SPSS, the Kolmogorov–Smirnov test was first used to determine whether the data were distributed normally or not. For quantitative variables with a normal distribution, the mean and standard deviation (SD) as well as the minimum (min)–maximum (max) values were reported, while for quantitative variables without a normal distribution, the median (*x̃*) and interquartile range (IQR) values were given. For categorical variables, the Chi‐square (*χ* ^2^) test was used to evaluate relationships or differences between groups. Because the AA genotype was present in fewer than five individuals in most study groups, the CA and AA genotypes were combined for statistical analyses to ensure sufficient sample size for meaningful comparisons. The non‐parametric Mann–Whitney *U* test was used to compare nonnormally distributed two groups, while the nonparametric Kruskal–Wallis test was used to compare three groups. Relationships between continuous variables were examined using Pearson correlation analysis. Logistic regression analysis was performed to assess the relationships between dependent and independent variables. Statistical significance was defined as a *p* value < 0.05.

## 3 Results

### 3.1 Socio‐Demographic Characteristics

A total of 562 people were included in the study: 114 with OUD (median age 30.0 years; 18 women and 96 men), 148 with MUD (median age 31.0 years; 22 women and 126 men), 150 people who used both opioids and methamphetamine (opioid–methamphetamine co‐use group) (median age 30.0 years; 21 women and 129 men) and 150 healthy controls (median age 32.0 years; 6 women and 144 men). Table 1 presents the socio‐demographic characteristics of the participants. Statistically significant differences were observed between individuals diagnosed with SUD and healthy controls in height and weight (*p* = 0.001), but not in age (adjusted *p* = 0.058). Post‐hoc analyses with Bonferroni correction revealed a statistically significant decrease in weight in SUD groups compared to healthy controls (adjusted *p* = 0.001). Among people who use opioids, people who used methamphetamine, and people who concurrently used opioids and methamphetamine, a significant difference was found in the age of first use (*p* = 0.012). Further post‐hoc analyses showed a significant difference between the MUD and OMCU groups (adjusted *p* = 0.02). Significant differences were also found between individuals diagnosed with SUD group and healthy controls in marital status (*p* = 0.001), living status (*p* = 0.001), education (*p* = 0.001), occupation (*p* = 0.001), military disciplinary history (*p* = 0.03), prison history (*p* = 0.001), and probation history (*p* = 0.001). Furthermore, a history of at least one unsuccessful buprenorphine treatment was reported by 75.44% (*n* = 86) of the OUD group and 68% (*n* = 102) of the OMCU group.

**TABLE 1:** Demographics of the groups included in the study.

Parameters	OUD (*n* = 114)		MUD (*n* = 148)		OMCU (*n* = 150)		Controls (*n* = 150)		*p* Value
Age (years) x̃ (IQR)	30.0 (26.0–34.0)		31.0 (26.0–35.0)		30.0 (27.0–33.0)		32.0 (27.0–40.0)		0.058
Weight (kg) x̃ (IQR)	70.0 (62.5–76.0)		67.0 (60.0–77.0)		66.0 (60.0–73.0)		80.5 (75.0–90.0)		0.001
Height (cm) x̃ (IQR)	174.0 (169.0–178.0)		173.0 (169.0–178.0)		173.0 (169.0–180.0)		178.0 (173.0–180.0)		0.001
The onset age of first substance use (years) x̃ (IQR)	18 (16.0–23.0)		19.5 (16.0–26.0)		17.0 (15.0–21.0)		–		**0.012**
**Education**	*n*	% frequency	*n*	% frequency	*n*	% frequency	*n*	% frequency	0.001
Primary	20	17.5	27	18.2	23	15.3	11	7.3	
Secondary	55	48.2	54	36.5	59	39.3	18	12.0	
High school	30	26.3	53	35.8	57	38.0	78	52.0	
Under‐graduate	7	6.1	8	5.4	8	5.3	42	28.0	
Graduate	—	—	1	0.7	—	—	1	0.7	
**Occupation**	*n*	% frequency (95% CI)	*n*	% frequency (95% CI)	*n*	% frequency (95% CI)	n	% frequency (95% CI)	0.001
Working	78	68,4	66	44,6	73	48,7	139	92,7	
Not working	34	29,8	77	52,0	74	49,3	11	7,3	
**Marital status**	*n*	% frequency (95% CI)	*n*	% frequency (95% CI)	*n*	% frequency (95% CI)	*n*	% frequency (95% CI)	0.001
Single	75	65.8	74	50.0	99	66.0	53	35.3	
Married	29	25.4	55	37.2	37	24.7	96	64.0	
Widow/divorced	8	7.0	14	9.5	11	7.3	1	0.7	
**Living status**	*n*	% frequency (95% CI)	*n*	% frequency (95% CI)	*n*	% frequency (95% CI)	*n*	% frequency (95% CI)	0.001
Alone	9	7.9	21	14.2	13	8.7	21	14.0	
Partner	2	1.8	3	2.1	8	5.3	—	—	
Spouse	24	21.1	40	27	26	17.3	91	60.7	
Family (mother, father, and siblings)	77	67.5	79	53.4	100	66.7	38	25.3	
**Military disciplinary history**	*n*	% frequency (95% CI)	*n*	% frequency (95% CI)	*n*	% frequency (95% CI)	*n*	% frequency (95% CI)	0.03
Present	13	11.4	22	14.9	27	18.0	9	6.0	
Absent	99	86.8	120	81.1	120	80.0	139	92.7	
**Prison history**	*n*	% frequency (95% CI)	*n*	% frequency (95% CI)	*n*	% frequency (95% CI)	*n*	% frequency (95% CI)	0.001
Present	58	50.9	63	42.6	86	57.3	4	2.7	
Absent	54	47.4	79	53.4	61	40.7	145	96.7	
**Probation history**	*n*	% frequency (95% CI)	*n*	% frequency (95% CI)	*n*	% frequency (95% CI)	*n*	% frequency (95% CI)	0.001
Present	47	41.2	59	39.9	68	45.3	1	0.7	
Absent	65	57.0	83	56.1	79	52.7	149	99.3	

Abbreviations: %, frequency; IQR, interquartile range; MUD, individuals with methamphetamine use disorder diagnosis; *n*, sample size; OMCU, individuals with opioid and methamphetamine co‐use disorder diagnosis; OUD, individuals with opioid use disorder diagnosis; x̃, median.

### 3.2 Ways of Using Opioid and/or Methamphetamine

Methods of substance use varied significantly among groups and are summarized as follows. Among individuals with OUD, the vast majority (90.4%, *n =* 103) used foil; 2.6% (*n =* 3) used injection; 1.8% (*n =* 2) used both foil and injection; and 0.9% (*n =* 1) used pipe. Among those with MUD, the majority (65.5%, *n =* 97) used a pipe; 9.5% (*n =* 14) used both a pipe and foil; 9.5% (*n =* 14) used injection; 5.4% (*n =* 8) used foil; and 0.7% (*n =* 1) used both a pipe and injection. Among those with OMCU, 88% (*n =* 132) used foil; 5.3% (*n =* 8) used injection; 2.7% (*n =* 4) used both foil and injection for opioid use. For methamphetamine use, 76.7% (*n =* 115) used a pipe; 10.7% (*n =* 16) used foil; and 4.7% (*n =* 7) used injection. Among those who used both opioids and methamphetamine, 27.3% (*n =* 41) used them on the same day but at different times; 34% (*n =* 51) used them on the same day and at the same time; 27.3% (*n =* 41) used them on different days; and 4.7% (*n =* 7) used one immediately before or after the other.

### 3.3 Genotype Distribution of the *FAAH* rs324420 Polymorphism

Table 2 shows the frequencies of genotypes and variant alleles of the *FAAH* rs324420 polymorphism in individuals with OUD, MUD, and OMCU, as well as healthy subjects. The frequency of the CC genotype was 63.2% (*n =* 72) in individuals with OUD, 75.7% (*n =* 112) in individuals with MUD, 71.3% (*n =* 107) in individuals with OMCU, and 64% (*n =* 96) in healthy individuals. The variant allele (A) frequencies were 21%, 13%, 16%, and 19% in OUD, MUD, OMCU, and controls, respectively. Genotype frequencies in all four groups were in Hardy–Weinberg equilibrium (HWE) (*p* > 0.05). Multinomial logistic regression analysis showed that there was no significant association between the *FAAH* rs324420 polymorphism and susceptibility to OUD and OMCU (*p* > 0.05). On the other hand, in the MUD group, the multinomial logistic regression analysis, after adjusting for age as a covariate, the CA genotype significantly reduced the risk of disease compared to the reference genotype (OR = 0.578, 95% CI: 0.343–0.973, adjusted *p* = 0.039).

**TABLE 2:** The frequencies of genotypes and variant alleles of the *FAAH* rs324420 polymorphism in individuals with OUD, MUD, OMCU, and controls.

*FAAH* rs324420 genotypes	OUD (*n* = 114)		MUD (*n* = 148)		OMCU (*n* = 150)		Controls (*n* = 150)	
	*n*	%	*n*	%	*n*	%	*n*	%
CC	72	63.2	112	75.7	107	71.3	96	64.0
CA	36	31.6	33	22.3	39	26.0	50	33.3
AA	6	5.3	3	2.0	4	2.7	4	2.7
Variant allele (A) frequency	21%		13%		16%		19%	
HWE	*χ* ^2^ = 0.285 *p* = 0.59		*χ* ^2^ = 0.09 *p* = 0.76		*χ* ^2^ = 0.039 *p* = 0.84		*χ* ^2^ = 0.707 *p* = 0.40	
Multinominal logistic regression (reference group = CC)	**AA** *p* = 0.297 OR = 2.00 95% CI (lower–upper): 0.54–7.35		**AA** *p* = 0.569 OR = 0.643 95% CI (lower–upper): 0.14–2.94		**AA** *p* = 0.88 OR = 0.89 95% CI (lower–upper): 0.22–3.69		‐	
	**CA** *p* = 0.879 OR = 0.96 95% CI (lower–upper): 0.57–1.63		**CA** *p* = **0.031** OR = 0.57 95% CI (lower–upper): 0.34–0.95		**CA** *p* = 0.163 OR = 0.70 95% CI (lower–upper): 0.42–1.15		‐	

Abbreviations: %, frequency; CI, confidence interval; HWE, Hardy–Weinberg equilibrium; MUD, individuals with methamphetamine use disorder diagnosis; *n*, sample size; OMCU, individuals with opioid and methamphetamine co‐use disorder diagnosis; OUD, individuals with opioid use disorder diagnosis.

### 3.4 Impulsiveness, Craving, Opioid Withdrawal, and the Intensity of Anxiety and Depressive Symptoms

Table 3 shows the scale scores for impulsiveness, craving, opioid withdrawal, and depressive and anxiety symptoms in individuals in the three SUD groups included in the study, as well as the *p* values from the statistical comparisons between the groups. As can be seen in Table 3, the intensity of depressive and anxiety symptoms and impulsiveness was significantly lower in the control group than in the other three substance‐using groups (*p* = 0.001). Although there was no significant difference in the intensity of depressive and anxiety symptoms between individuals who use opioids and/or methamphetamine (*p* > 0.05), the lowest scores were observed in the OMCU group. In the three groups that used substances, the median withdrawal scale score was 3.0 (Table 3). There was a significant difference between opioid and/or methamphetamine users in terms of craving (*p* = 0.021). In the paired group comparisons, craving scale scores were higher among individuals who use opioids and/or methamphetamine (20.0; 13.0–28.0) than among people who only use opioids (18.0; 14.0–28.0) (*p* = 0.009).

**TABLE 3:** Scale scores of the groups included in the study.

Scales	OUD (*n* = 114)	MUD (*n* = 148)	OMCU (*n* = 150)	Controls (*n* = 150)	*p* Value
**Impulsiveness (BIS‐11)** x̃ **(IQR)**	31.0 (25.0–37.0)	32.0 (27.0–38.0)	32.0 (26.0–37.0)	20 (16.0–25.0)	0.001
**SCS** x̃ **(IQR)**	18 (14.0–28.0)	17.5 (8.3–25.0)	20 (13.0–28.0)	—	0.021
**COWS** x̃ **(IQR)**	3.0 (1.0–6.0)	—	3.0 (1.0–7.0)	—	0.519
**BDI‐II** **Mean ± SD** **(min.–max.)**	29.6 ± 13.0 (2.0–61.0)	29.3 ± 16.4 (0.0–74.0)	28.2 ± 12.1 (6.0–63.0)	5.5 ± 5.9 (0.0–28.0)	0.001
**BAI** x̃ **(IQR)**	20.0 (10.0–31.0)	20.0 (7.0–33.5)	17.5 (9.0–29.3)	1.0 (0.0–4.0)	0.001

Abbreviations: BAI, Beck Anxiety Inventory; BDI, Beck Depression Inventory; BIS, Barratt Impulsiveness Scale; COWS, Clinical Opiate Withdrawal Scale; IQR, interquartile range; MUD, individuals with methamphetamine use disorder diagnosis; *n*, sample size; OMCU, individuals with opioid and methamphetamine co‐use disorder diagnosis; OUD, individuals with opioid use disorder diagnosis; SD, standard deviation; SCS, substance craving scale; x̃, median.

### 3.5 The Intensity of Anxiety and Depressive Symptoms, Opioid Withdrawal, Craving and Impulsiveness, Across *FAAH* rs324420 Genotypes

In this study, *FAAH* rs324420 genotypes were compared to determine whether differences existed in scale scores. As the frequency of the AA genotype was fewer than five in most of the study groups, individuals with AA and CA genotypes were combined and statistically compared with individuals with the CC genotype. No significant differences were observed between the *FAAH* rs324420 genotypes with regard to depressive and anxiety symptoms, opioid withdrawal, craving, or impulsiveness in any of the four groups (*p* > 0.05). Although no statistically significant differences were found between *FAAH* rs324420 genotypes in individuals who use only methamphetamine (*p* > 0.05), depressive symptoms, craving, and anxiety tended to be higher in those with the CC genotype compared to those with the CA + AA genotype (Table 4).

**TABLE 4:** Comparison of genotypes of the *FAAH* rs324420 polymorphism of the SUD groups in terms of impulsiveness, craving, opioid withdrawal, and depressive and anxiety symptoms.

Scales	OUD (*n* = 114)		MUD (*n* = 148)		OMCU (*n* = 150)	
	CC (*n* = 72)	CA+AA (*n* = 42)	CC (*n* = 114)	CA+AA (*n* = 38)	CC (*n* = 107)	CA+AA (*n* = 43)
**Impulsiveness (BIS)** **Median (IQR)**	31.0 (25.0–37.0)	31.5 (28.0–36.25)	32 (28.0–38.3)	33 (25.0–36.3)	32 (26.0–39.0)	31 (25.0–34.3)
*p* Value	*p* = 0.257		*p* = 0.448		*p* = 0.087	
**SCS** **Median (IQR)**	18.0 (13.8–27.3)	18.5 (13.8–29.3)	18.5 (8.8–26.0)	15.5 (7.5–22.5)	21 (14.0–27.0)	18 (11.5–30.0)
*p* Value	*p* = 0.572		*p* = 0.389		*p* = 0.683	
**COWS** **Median (IQR)**	3.0 (0.3–6.8)	3.0 (1.0–5.0)	3 (1.0–7.0)	2 (1.0–6.0)	3 (1.0–7.0)	3 (1.0–7.0)
*p* Value	*p* = 0.813		*p* = 0.572		*p* = 0.851	
**BDI‐II** Mean ± SD (min.–max.)	29.22 ± 12.15 (2.0–60.0)	30.20 ± 14.55 (4.0–61.0)	30.4 ± 16.7 (0.0–74.0)	25.9 ± 15.5 (0.0–59.0)	28.5 ± 11.8 (7.0–60.0)	27.31 ± 12.77 (6.0–63.0)
*p* Value	*p* = 0.707		*p* = 0.166		*p* = 0.581	
**BAI** **Median (IQR)**	19.5 (10.0–30.8)	21.5 (10.0–34.5)	23.5 (8.0–35.8)	16.0 (4.0–28.0)	17.0 (9.0–29.0)	18.0 (8.0–30.0)
*p* Value	*p* = 0.392		*p* = 0.112		*p* = 0.859	

Abbreviations: BAI, Beck Anxiety Inventory; BDI‐II, Beck Depression Inventory II; BIS, Barratt Impulsiveness Scale; COWS, Clinical Opiate Withdrawal Scale; IQR, interquartile range; MUD, individuals with methamphetamine use disorder diagnosis; *n*, sample size; OMCU, individuals with opioid and methamphetamine co‐use disorder diagnosis; OUD, individuals with opioid use disorder diagnosis; SCS, Substance Craving Scale.

### 3.6 Quantity of Opioid/Methamphetamine Consumed, Duration of Opioid/Methamphetamine Use, and Age Onset of First Opioid/Methamphetamine Use, Across FAAH rs324420 Genotypes

There were no significant differences between *FAAH* rs324420 genotypes in the amount of opioid ***/*** methamphetamine used, duration of opioid ***/*** methamphetamine use, and age at onset of first opioid use among individuals who use opioid and/or methamphetamine (Table 5) (*p* > 0.05).

**TABLE 5:** Comparison of genotypes of the *FAAH* rs324420 polymorphism in terms of the amount of opioid ***/*** methamphetamine used, duration of opioid ***/*** methamphetamine use, and age of onset of first substance use.

Parameters	OUD (*n* = 114)		MUD (*n* = 148)		OMCU (*n* = 150)	
	CC (*n* = 72)	CA + AA (*n* = 42)	CC (*n* = 114)	CA + AA (*n* = 38)	CC (*n* = 107)	CA + AA (*n* = 43)
**The onset age of first substance use (years)** **Median (IQR)**	19.0 (16.0–24.0)	18.0 (16.0–20.8)	20.0 (16.0–27.0)	18.0 (16.0–23.0)	17.0 (15.0–20.0)	18.0 (15.0‐24.0)
*p* Value	*p* = 0.499		*p* = 0.210		*p* = 0.225	
**Duration of heroin use (years)** **Median (IQR)**	8.0 (6.0–12.0)	8.0 (5.0–10.0)	—		10.0 (4.5–13.0)	8.0 (5.0–12.0)
*p* Value	*p* = 0.440		—		*p* = 0.774	
**Quantity of heroin consumed (g/day)** **Median (IQR)**	2.0 (1.0–4.5)	3.0 (1.5–4.88)	—		2.5 (1.0–5.0)	2.5 (1.5–4.0)
*p* Value	*p* = 0.547		—		*p* = 0.849	
**Duration of Meth use (years)** **Median (IQR)**	—		2.0 (1.0–4.0)	2.0 (1.0–3.5)	2.0 (1.0–4.0)	1.25 (0.5–6.0)
*p* Value	—		*p* = 0.451		*p* = 0.310	
**Quantity of Meth consumed (g/day)** **Median (IQR)**	—		1.0 (1–2)	1.0 (1–2)	1.0 (0.5–1.0)	1.0 (0.5–1.0)
*p* Value	—		*p* = 0.296		*p* = 0.536	

Abbreviations: IQR, interquartile range; MUD, individuals with methamphetamine use disorder diagnosis; *n*, sample size; OMCU, individuals with opioid and methamphetamine co‐use disorder diagnosis; OUD, individuals with opioid use disorder diagnosis.

### 3.7 Correlation Analysis

In individuals diagnosed with OUD, a positive and significant relationship was found between the duration of opioid use and craving (*r* = 0.244; *p* = 0.012); between the quantity of opioid consumed and opioid withdrawal (*r* = 0.213; *p* = 0.025), depressive symptoms (*r =* 0.229; *p* = 0.016), anxiety (*r =* 0.307; *p* = 0.001); between opioid withdrawal and depressive symptoms (*r =* 0.225; *p* = 0.018) and anxiety (*r =* 0.424; *p* = 0.001); between depressive symptoms and both craving (*r =* 0.254; *p* = 0.008), and impulsiveness (*r =* 0.319; *p* = 0.001). A negative and significant correlation was found between the age of onset of substance use and the daily amount of opioid used (*r =* −0.214; *p* = 0.028). In individuals who use methamphetamine (*n* = 148), a positive and significant relationship was found between the duration of methamphetamine use and craving (*r =* 0.199; *p* = 0.028); between the quantity of methamphetamine consumed and both anxiety (*r =* 0.241; *p* = 0.004), and impulsiveness (*r =* 0.177; *p* = 0.037); and between depressive symptoms and craving (*r =* 0.356; *p* = 0.001), impulsiveness (*r =* 0.351; *p* = 0.001), and anxiety (*r =* 0.680; *p* = 0.001). A negative and significant correlation was found between the age at onset of substance use and the daily amount of methamphetamine used (*r =* −0.180; *p* = 0.034), as well as between the duration of methamphetamine use and both anxiety (*r =* −0.183; *p* = 0.03) and craving (*r =* −0.178; *p* = 0.048). Among those diagnosed with OMCU, a positive and significant correlation was found between the amount of methamphetamine used and the amount of opioid used (*r =* 0.291; *p* = 0.001); between the duration of opioid use and the duration of methamphetamine use (*r =* 0.298; *p* = 0.001).

## 4 Discussion

In this study, we investigated psychological characteristics and genetic polymorphisms in individuals with opioid use disorder (OUD), methamphetamine use disorder (MUD), and opioid–methamphetamine co‐use (OMCU). We found that people in the OMCU group had significantly higher craving and withdrawal scores compared to those with OUD only. Impulsivity and aggression levels were also elevated among co‐users, highlighting the complex clinical profile of this group. Regarding genetic findings, the CA genotype was associated with a significantly reduced risk of disease compared to the reference genotype. Additionally, a high proportion of participants reported at least one unsuccessful buprenorphine treatment attempt, particularly in the OUD group. These findings together emphasize the clinical and genetic factors that may contribute to the challenges in treating opioid and methamphetamine co‐use.

FAAH, which has long been a target of research on substance use disorders, has more recently become a particular focus of study. The results of these studies have shown that genetic variations in the *FAAH* gene are associated with, and may contribute to, substance use disorder (Chiang et al. 2004; López‐Moreno et al. 2012; Sipe et al. 2002). From a pharmacological and therapeutic perspective, it has been suggested that inhibition of FAAH could reduce or reverse the negative effects experienced during withdrawal syndromes. FAAH inhibition is therefore thought to also reduce drug‐seeking behavior in individuals (Niemela and Terry 2023).

The endocannabinoid system, including FAAH, is associated with brain regions involved in reward, avoidance, mood, and the development of substance use disorders. Molecules that regulate the endocannabinoid system are therapeutic targets for neuropsychiatric disorders and neurodegenerative diseases such as Alzheimer's disease and Parkinson's disease (Ren et al. 2020). Rimonabant, a CB1 receptor antagonist, was initially promising for SUD and obesity, but was discontinued due to side effects such as depression and suicidal tendencies. Treatment interest has shifted to FAAH and MAGL inhibitors, FABP inhibitors, and nonaddictive CB1 or CB2 phytocannabinoids (Galaj and Xi 2019). These inhibitors have fewer side effects and modulate the hypothalamic‐pituitary‐adrenal axis through antidepressant and anxiolytic mechanisms of action, as well as regulating synaptic and neuronal plasticity (Ren et al. 2020).

The *FAAH* rs324420 (Pro129Thr or C385A) polymorphism results in a substitution of proline (Pro/C allele) to threonine (Thr/A allele) in the FAAH protein (Sipe et al. 2002). It has been reported that this variant leads to reduced enzyme catalytic activity and protein stability in cell lines and rodents, resulting in increased levels of anandamide in the brain (Chiang et al. 2004; Dincheva et al. 2015). Another study reported increased levels of AEA and 2‐AG, with almost half the enzymatic activity of FAAH observed in individuals carrying this polymorphism compared to those not carrying the variant (Chiang et al. 2004). Several studies have reported that the rs324420 polymorphism may be associated with addiction to substances such as cocaine, alcohol, cannabis, heroin, nicotine, and other drugs (Chiang et al. 2004; López‐Moreno et al. 2012; Sipe et al. 2002). In a study in which the *FAAH* rs324420 polymorphism was genotyped and fMRI (functional magnetic resonance imaging) data were collected from individuals, it was observed that individuals with the CC genotype exhibited increased activation in the brain's reward circuit areas compared to those with the AA genotype (Filbey et al. 2010). In another study using imaging genetics, it was found that participants carrying the A allele of *FAAH*, which is associated with decreased enzyme expression and increased anandamide signaling, showed reduced amygdala reactivity in response to fear and anxiety in comparison to individuals with the CC genotype. Furthermore, individuals with the A allele have been shown to exhibit heightened ventral striatal reactivity and impulsivity related to reward, in comparison to those who carry the CC genotype (Hariri et al. 2009). Dincheva et al. (2015) conducted a study in which they observed that human and mouse carriers of the A allele of the *FAAH* gene exhibited reduced levels of fear and anxiety. A further study reported that individuals carrying the *FAAH* rs324420 A allele are more likely to experience “anxiety and depression” (Lazary et al. 2016). It was hypothesized that individuals carrying the *FAAH* rs324420 A allele may have a congenitally impaired endocannabinoid system. It was reported that the impaired endocannabinoid system could eliminate the “feeling of well‐being” in individuals, leading to substance use disorder (Bornscheuer et al. 2023). In the current study, a logistic regression analysis showed that the CA genotype of the *FAAH* rs324420 may have a protective effect compared with the AA or CC genotypes only in the group of methamphetamine users. It is plausible that a reduction in FAAH activity may enhance endocannabinoid signaling and modulate dopamine‐related pathways, thereby attenuating the reinforcing effects of methamphetamine. It is interesting to note that the protective effect of the CA genotype was not observed in individuals with OUD or in opioids and methamphetamine co‐users, suggesting the hypothesis that different types of substances, either alone or in combination, may engage distinct neurobiological mechanisms. However, there is a need for further studies to validate this finding in larger cohorts and to elucidate the underlying biological mechanisms.

As previously discussed, the CA genotype may be protective in methamphetamine users. In parallel with this finding, it was determined that individuals with the CC genotype exhibited higher levels of depressive symptoms, craving, and anxiety compared with those with the CA+AA genotype. This result suggests that *FAAH* rs324420 may contribute to methamphetamine use both directly and indirectly by influencing psychological factors associated with substance use disorder (i.e., depression, craving, and anxiety). It may be hypothesized that the CC genotype could lead to a decrease in anandamide levels through an increase in FAAH activity. This, in turn, could result in the exacerbation of symptoms associated with anxiety and depression.

A study conducted in a Japanese population indicated that the *FAAH* rs324420 polymorphism is not significantly associated with methamphetamine use disorder and psychosis. Furthermore, no substantial correlation was identified between this polymorphism and characteristics such as the age of onset of methamphetamine use, the duration until the onset of psychosis, prognosis, spontaneous relapse, and multiple substance abuse (Morita et al. 2005). In contrast, a study of individuals from four distinct ethnic groups (Malay, Chinese, Kadazan–Dusun, and Bajau) revealed a significant and strong association between the A allele in this polymorphism (compared to the control group) and the propensity for methamphetamine use disorder, particularly among the Malay and Chinese populations (Sim et al. 2013). Another study supporting these findings examined the effects of *FAAH* mRNA and protein levels and the *FAAH* rs324420 polymorphism on methamphetamine use disorder in the Chinese Han population. The study found that the A allele of the *FAAH* rs324420 polymorphism (AA, AC, and AC/AA) significantly increased the risk of methamphetamine use disorder (Zhang et al. 2020). When the findings from studies conducted in Asian populations were considered alongside our study in Caucasians, the results supported the notion that the effects of alleles may differ across populations.

According to the TUBIM (2023) report, the age at which drug use starts is between 15 and 24 years old in Turkey. In the current study, the average age at onset of drug use was 18.0 years for those with OUD, 20.0 years for those with MUD and 17.0 years for those with OMCU. Analyses showed that there was a significant relationship between the MUD and OMCU groups in terms of age onset of first use. The fact that the median age onset of first use was 17.0 years for opioid and methamphetamine co‐users, and 20.0 years for those who used methamphetamine alone. This finding suggests that those who start using drugs at an early age may be more prone to polydrug use in the future.

Opioid and methamphetamine co‐users demonstrated a higher prevalence of craving scale scores (20.0; 13.0–28.0) in comparison to people who only use opioids (18.0; 14.0–28.0) (*p* = 0.009), indicating that opioid and methamphetamine co‐users exhibit a heightened craving for the substance in comparison to those who use opioids only, and that polydrug use may further complicate the addiction process. Opioids have a generally depressant effect on the central nervous system, producing sensations of relaxation and sedation (Young‐McCaughan and Miaskowski 2001). In contrast, methamphetamine has a stimulant effect (Zapata et al. 2021). These two effects, which are diametrically opposed, have the capacity to increase craving levels by affecting the brain's reward system in a more complex way (Li et al. 2015; Moreno‐Rius and Miquel 2017; Pattison et al. 2012). Wang et al. (2017) reported that the addiction process of individuals with OUD was shorter than that of individuals with MUD, with shorter transitions from the onset of drug use to the first drug craving (19.5 vs. 50.0 days), regular use (30.0 vs. 60.0 days), and compulsive use (50.0 vs. 85.0 days). However, no significant differences in the addiction process were observed in the frequency of drug administration, except that individuals with OUD reported more administrations of the drug (20.0 vs. 15.0) before progressing to the stage of compulsive drug use. The mentioned study was conducted with individuals who used methamphetamine and opioids independently (Wang et al. 2017). In our study, however, compared to the people who only use opioids, the craving effect in the co‐use of these substances might potentially be amplified by methamphetamine.

A number of studies have demonstrated that psychological disorders in individuals may be triggered by substance use or that existing symptoms may be exacerbated. This phenomenon frequently results in individuals committing criminal acts (Neighbors et al. 1992). The results of a meta‐analysis study, which examined approximately 30 articles involving adults, children, and adolescents, indicated that individuals who use substances are 3–4 times more likely to commit crimes (e.g., robbery, theft) than those who do not use substances (Bennett et al. 2008). Another study supporting this perspective reported that 32.8% of adolescents committed crimes, resulting in their arrest or conviction while under the influence of substances (Ögel and Aksoy 2007). In a study conducted by Sutherland et al. (2015), it was observed that 71% (630) of 887 individuals committed theft, and 73% (647) committed violent crimes. It was reported that 24% (151) of individuals who committed theft used methamphetamine and 29% (183) used benzodiazepines, while 32% (207) of individuals who committed violent crimes used opioids and 32% (207) used alcohol. The findings of the preliminary studies have indicated the presence of a correlation between the substance utilized and the perpetrated crime (Sutherland et al. 2015). In the present study, a significant difference was found between healthy individuals who had not used psychoactive substances other than cigarettes throughout their lives and individuals who had used opioids and/or methamphetamine in terms of prison history and probation history. This finding is in parallel with those of previous studies.

This study is one of the first to investigate the role of the *FAAH* rs324420 polymorphism in individuals with OUD, MUD, and especially in those with concurrent opioid–methamphetamine use in a Turkish population. The relatively large sample size and the inclusion of both clinical and healthy control groups strengthen the reliability of the findings. Another strength is the use of validated psychometric scales to assess impulsivity, craving, anxiety, and depressive symptoms, which allowed for a comprehensive evaluation of clinical characteristics. However, several limitations should be noted. First, the cross‐sectional design does not allow for causal inferences. Second, the study was conducted in a single treatment center, which may limit the generalizability of the results. Third, although RFLP genotyping was partially confirmed by sequencing, not all samples were sequenced. Additionally, the lack of a methamphetamine craving scale validated in Turkish represents a limitation in the assessment of craving in MUD participants. Finally, potential confounding variables such as environmental factors and co‐occurring psychiatric conditions were not fully controlled. Future multicenter and longitudinal studies with larger and more diverse populations are needed to validate and extend these findings.

In conclusion, the present study demonstrated that the *FAAH* rs324420 polymorphism is associated with an elevated risk of methamphetamine use disorder in the Turkish population, both directly and by influencing physiological characteristics related to substance use disorder. However, this polymorphism showed no significant effect on OUD or on the co‐use of opioids and methamphetamine. To consider the use of FAAH inhibitors in the treatment of methamphetamine use disorder, it is important to replicate the findings of this study in larger samples and in other populations.

## Author Contributions

**Özlem Erekli‐arat**: conceptualization, methodology, investigation, writing – original draft. **Meltem Tepe**: methodology, resources. **Selin Özkan‐kotiloğlu**: investigation, methodology, formal analysis. **Mustafa Danışman**: resources. **Dilek Kaya‐akyüzlü**: conceptualization, investigation, funding acquisition, writing – review and editing, methodology, formal analysis, project administration, supervision.

## Funding

This study was supported by the Ankara University Scientific Research Projects Coordination Unit (grant number: TDK‐2023‐3094 awarded to Dilek Kaya‐Akyüzlü).

## Ethics Statement

Our study was carried out in accordance with the Ankara University Faculty of Medicine Human Research Ethics Committee (approval number: I03‐109‐22 on March 10, 2022) and the Helsinki Declaration.

## Consent

Written informed consent was obtained from all study participants.

## Conflicts of Interest

The authors declare no conflicts of interest.

## Data Availability

The data supporting the conclusions of this study are accessible from the corresponding author upon submission of a reasonable request.
